# Severe Aortic Stenosis Associated with Other Valve Diseases: Open Surgery or Percutaneous Treatment?

**DOI:** 10.31083/j.rcm2503099

**Published:** 2024-03-08

**Authors:** Sergio Moral, Marc Abulí, Esther Ballesteros, Pau Vilardell, Laura Gutiérrez, Ramon Brugada

**Affiliations:** ^1^Cardiology Department, Hospital Universitari Doctor Josep Trueta, 17007 Girona, Spain; ^2^Centro de Investigación Biomédica en Red de Enfermedades Cardiovasculares (CIBERCV), 28029 Madrid, Spain; ^3^Department of Medical Sciences, Universitat de Girona, 17003 Girona, Spain; ^4^Dirección Territorial de Radiología y Medicina Nuclear de Girona. IDI. IDIBGI, 17007 Girona, Spain

**Keywords:** aortic stenosis, valvular heart diseases, transcatheter aortic valve replacement, mitral regurgitation, tricuspid regurgitation, mitral stenosis

## Abstract

Treatment decisions in the context of severe aortic stenosis (AS) associated with other valvular heart diseases (VHDs) have become a major challenge in recent years. Transcatheter aortic valve replacement (TAVR) in AS has increased significantly in younger patients with lower surgical risk, which has complicated the choice of the best treatment in cases of other associated valvulopathies. The most frequently associated lesions in this clinical scenario are mitral regurgitation (MR), mitral stenosis, and tricuspid regurgitation (TR). Furthermore, it should be noted that different percutaneous techniques are now available to accommodate any associated valvulopathies, which has considerably broadened the range of therapeutic options. The management of AS treated in isolation, especially by TAVR, has also shown that many cases of significant MR or TR are substantially reduced without any intervention. However, although some parameters have been described as potential risk factors in predicting the poor outcome of untreated VHDs, which cases will progress in a clinically more aggressive way remains uncertain. This review aimed to evaluate the most recent publications to provide the pathophysiology and prognosis of severe AS associated with other significant VHDs and to evaluate the best invasive therapeutic approach depending on the associated valvular disease.

## 1. Introduction

In the context of multiple-valve disease, invasive treatment of severe aortic stenosis (AS) has changed considerably in recent years [1, 2, 3, 4]. Classically, surgery has been the treatment of choice in patients with significant combined valvular heart disease (VHD) and AS. However, following the development of percutaneous techniques, this approach has evolved dramatically in such cases [1, 2, 3, 4]. Nowadays, surgical, percutaneous, or hybrid therapy can all be considered for the same patient, depending on the type of lesions and characteristics the patient exhibits [1, 5, 6]. Furthermore, each procedure has multiple options and types of implantable prostheses [7, 8, 9]. Transcatheter aortic valve replacement (TAVR) has greatly impacted AS management, is currently the most widespread percutaneous valve therapy, and, in some countries, has already surpassed surgical treatment in terms of the number of procedures [10, 11, 12].

According to the latest published guidelines, recommendations for interventional treatment of severe isolated AS are generally surgical in patients <75 years or with low interventional risk (STS-PROM/EuroScore II values <4%) [5, 6]. Conversely, in cases where the age of the patient is ≥75 years, or they are high risk (Society of Thoracic Surgeons Predicted Risk of Mortality score (STS-PROM)/EuroScore II values >8%), TAVR is indicated [5, 6]. Therefore, there is a surgical risk range (between 4% and 8%) in which the invasive management is not clearly defined. Furthermore, in patients with other associated significant valvulopathies, the recommendations are more open and allow for greater freedom of choice by the Heart Team at each center, according to their experience [1, 5, 6]. Moreover, in cases where the surgical approach is not a suitable option, percutaneous treatment should be considered for AS and the other involved lesions. However, this management could be performed via different procedures and by assessing the clinical impact of the treatment in each of its phases [3, 4, 5, 6].

Although severe AS has been reported in other valve diseases, some associations are more prevalent than others in clinical practice. Mitral regurgitation (MR), tricuspid regurgitation (TR), and mitral stenosis (MS) are the most frequently involved lesions in cases of aortic multiple-valve disease [13, 14, 15].

This review aimed to describe the pathophysiology and prognosis of severe AS associated with other significant VHDs and to use recent publications to determine the best invasive therapeutic approach depending on the associated valvular disease.

## 2. Pathophysiology and Prognosis of Lesion Association

The VHDs most frequently associated with AS are MR, MS, and TR, and these scenarios form the main focus of this review [13, 14, 15].

### 2.1 Severe AS Associated with MR

**Fig. 1.**
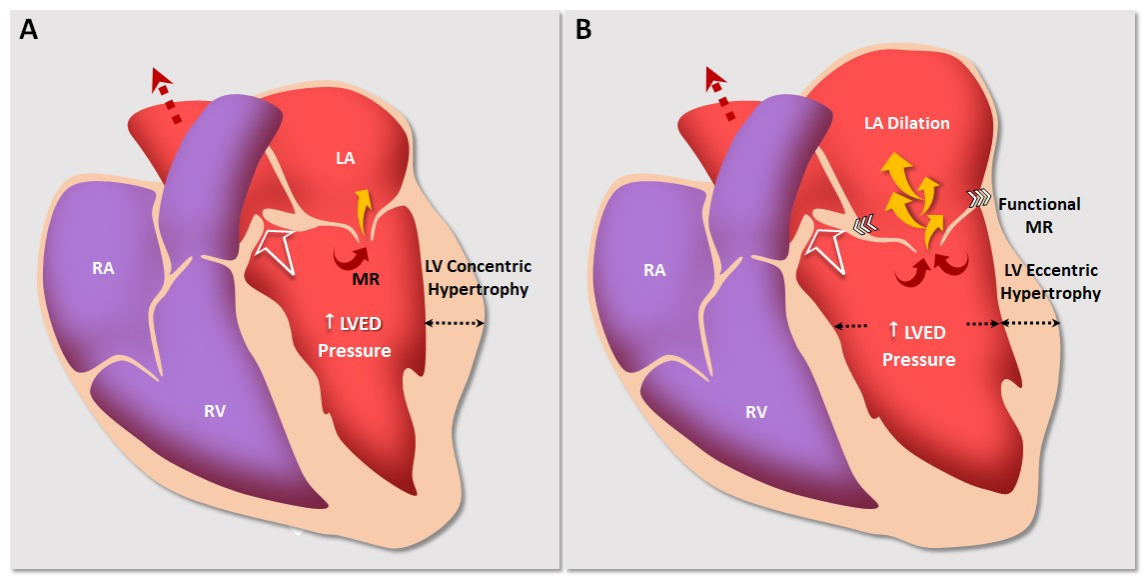
**Pathophysiological mechanisms of generating functional MR secondary to AS**. (A) First stage of the disease with increased left ventricular end-diastolic pressure and ventricular concentric hypertrophy. The white arrow indicates the flow through the left ventricular outflow tract. The black discontinuous arrow exhibits the flow through the ascending aorta. This pressure overload generates initial functional mitral regurgitation (yellow arrow). (B) Second stage with progressive atrial and mitral valve annulus dilation (horizontal white arrows). These hemodynamic changes provoke mitral regurgitation to increase progressively. LA, left atrium; LV, left ventricle; LVED pressure, left ventricular end-diastolic pressure; RA, right atrium; RV, right ventricle; MR, mitral regurgitation; AS, aortic stenosis.

This association is frequent, and it has been reported that up to 20% of patients referred for TAVR present with moderate/severe MR [2, 16]. The involvement of both valve diseases is the result of three main reasons. First is the incidence of MR in the general population. Since MR represents prevalent valvulopathy (between 2–3% of the population presents a significant MR) [17], both lesions tend to overlap frequently in the same patient. Second, both valvulopathies share common etiologies, such as degenerative disease, increasing their combined incidence [18, 19]. Moreover, other illnesses can also involve both lesions: congenital diseases (bicuspid aortic valve) [20], infiltrative diseases (amyloidosis, Fabry disease, etc.) [21, 22], endocarditis [23, 24], etc. However, the latter of these tend to be more uncommonly associated with degenerative diseases in developed countries. Finally, the pathophysiology of AS also impacts the generation of MR (Fig. 1). Pressure overload, secondary to severe AS, causes left ventricular hypertrophy, increased left ventricular end-diastolic pressure, diastolic dysfunction, and left atrial dilation in the early stages of the disease [25, 26]. These morphological and hemodynamic changes can provoke a functional MR mechanism or cause it to increase in instances where it already exists. It usually worsens as the illness progresses, and according to estimates, up to 60% of patients with severe AS have some degree of functional MR [27].

Regarding the prognosis conferred by significant MR in cases with severe AS, studies are consistent in the clinical impact this entails. The concomitance of moderate/severe MR increases short- and long-term mortality in patients receiving treatment for AS [1, 2, 28, 29]. Although most series suggest that MR organic etiology could be an important prognostic factor compared to predominantly functional cases, there is no definitive consensus in the published literature [30, 31, 32, 33]. However, it seems reasonable to consider that an organicity in the pathophysiological mechanism involves a potential substrate that may have a greater impact on the patient’s prognosis, at least in the long term. When the MR degree is at its most moderate (moderate or less), studies disagree about the real prognostic significance of this condition [33, 34]. Furthermore, it is noteworthy that after aortic valve treatment, more than 50% of cases significantly reduce the degree of MR at the 1-year follow-up, regardless of the etiological mechanism [32]. This fact makes it even more difficult to determine the real clinical impact of cases with lower grades of MR.

### 2.2 AS Associated with MS

**Fig. 2.**
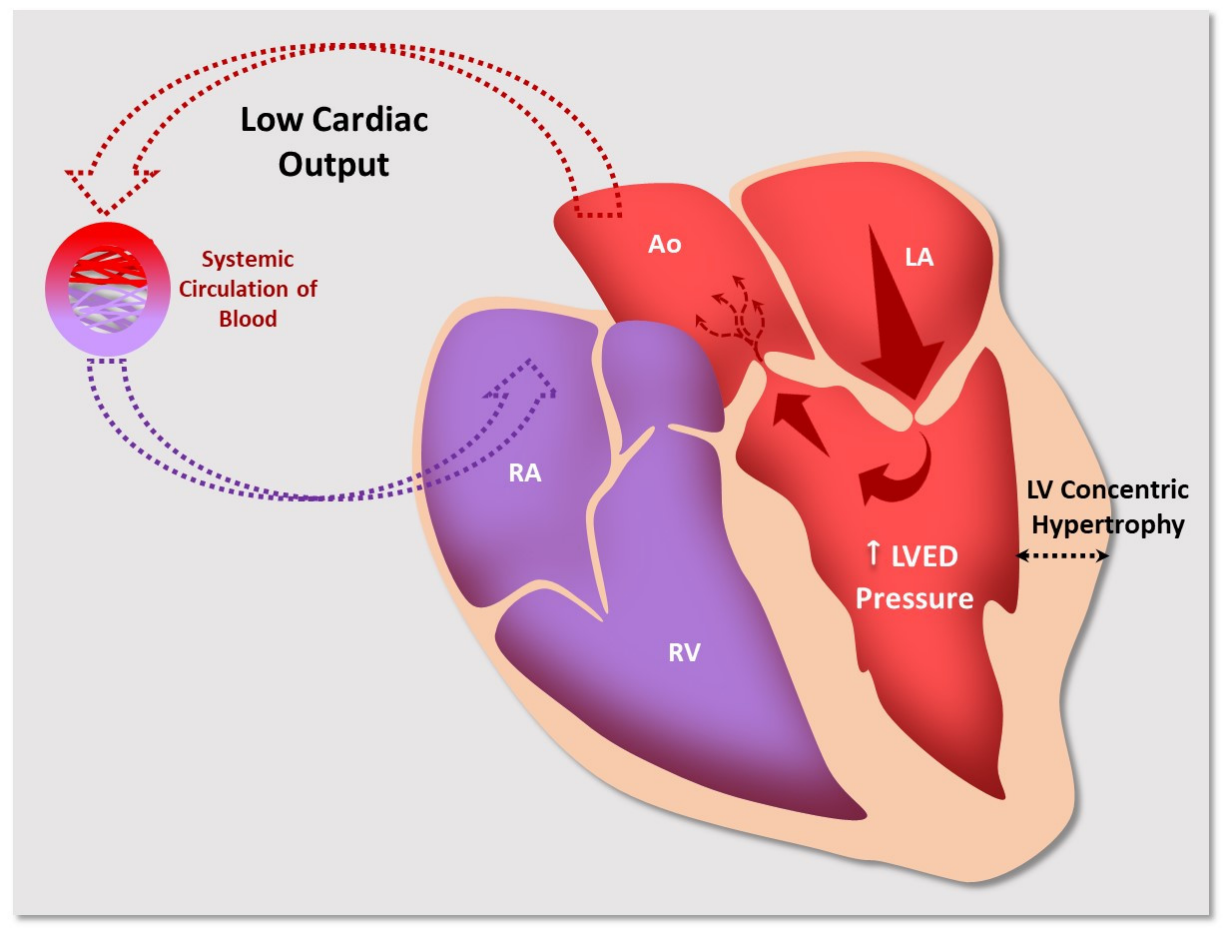
**Pathophysiological mechanism of poor clinical tolerance, generated by hemodynamic compromise, resulting from the concomitant presence of MS and AS**. Blood flow is compromised by both valvular stenoses and results in decreased cardiac output (red arrows represent cardiac output that becomes progressively smaller). The discontinuous red arrow indicates cardiac flow to peripheral tissues, and the discontinuous blue arrow depicts systemic venous flow. LA, left atrium; LV, left ventricle; LVED pressure, left ventricular end-diastolic pressure; RA, right atrium; RV, right ventricle; Ao, aorta; MS, mitral stenosis; AS, aortic stenosis.

Between 12% to 18% of patients undergoing TAVR have some degree of MS, while it is severe in 2–3% [35, 36, 37]. Classically, the etiology involving both VHDs was rheumatic [18, 19]. Nevertheless, in recent years, as the population ages, the degenerative cause is significantly increasing this association in developed countries [35]. A combination of MS and AS can worsen the symptomatology and hemodynamics of patients, especially because of the decrease in cardiac output (Fig. 2) [13]. Nonetheless, unlike MR, the degree of MS severity is not directly affected by AS pathophysiology since the effective orifice of the mitral valve remains unchanged. Conversely, some parameters in the assessment of MS, such as transvalvular gradient, could be influenced by the AS presence due to the impairment of the left ventricular diastolic function and the increase in left ventricular end-diastolic pressure [13, 15]. Therefore, anatomical evaluation of the mitral valve orifice using planimetry and imaging techniques is critical to accurately estimating the MS degree [1, 13].

Regarding prognosis, a disparity in the definition of stenosis severity exists among the studies. While some consider severe MS with valvular areas ≤1.5 ${\mathrm{cm}}^{2}$ or mean transvalvular gradients ≥5 mmHg, others are more restrictive and place cut-off points at values of ≤1.0 ${\mathrm{cm}}^{2}$ or ≥10 mmHg, respectively [1, 2, 35, 36, 37]. Most authors agree that significant MS worsens the prognosis of patients treated with severe AS, especially after one year of follow-up, mainly in cases with a valvular area ≤1.0 ${\mathrm{cm}}^{2}$ [1, 2, 35, 36, 37]. Furthermore, Asami *et al*. [35] reported that the risk of a disabling stroke was three times higher in patients with some degree of MS during the first year after TAVR compared to those without any stenosis. Although this fact could be secondary to an increased risk of embolic complications in patients with MS during and after the invasive procedure, given that the majority of events occurred during the first 30 days post-intervention, it is certainly an added risk to consider in the planning of aortic valve procedures.

### 2.3 AS Associated with TR

**Fig. 3.**
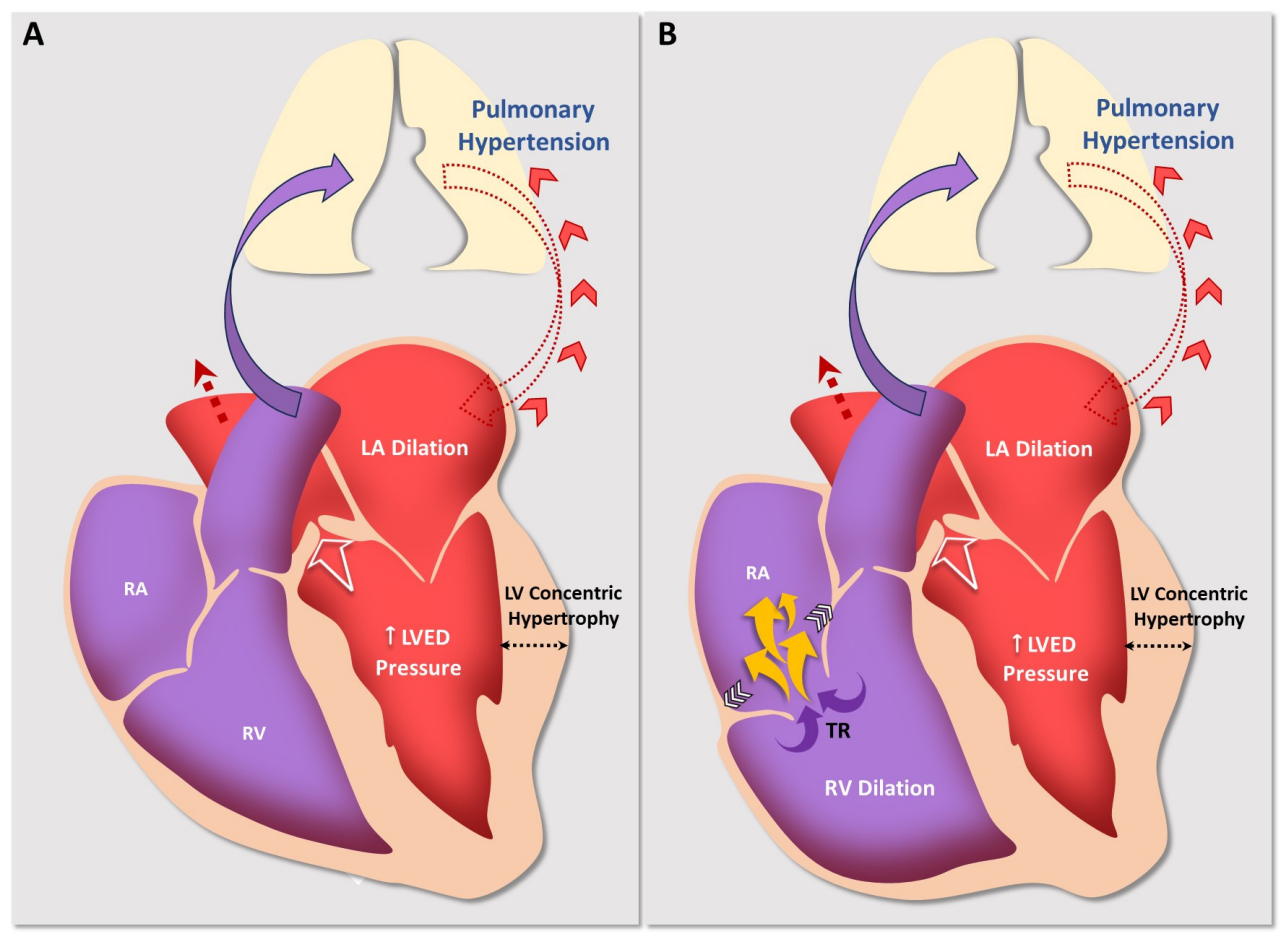
**Pathophysiological mechanisms in the generation of ventricular secondary TR caused by AS**. (A) First stage of the disease with increased left ventricular end-diastolic pressure and pulmonary hypertension. The blue arrow indicates anterograde pulmonary flow. The red discontinuous arrow between the lung and LA depicts anterograde flow through pulmonary veins, while the red arrowheads indicate post-capillary pulmonary hypertension, secondary to AS. (B) Second stage with an increase in post-capillary pulmonary hypertension and subsequent right overload, with dilation (white horizontal arrows) or dysfunction in the right ventricle. These changes lead to an increase in ventricular secondary TR (yellow arrows). LA, left atrium; LV, left ventricle; LVED pressure, left ventricular end-diastolic pressure; RA, right atrium; RV, right ventricle; TR, tricuspid regurgitation; AS, aortic stenosis.

AS is frequently associated with a minimum TR rate of moderate grade, which is reported in 11–27% of patients who undergo TAVR [1, 2, 36, 38, 39]. The incidence of both VHDs in the same patient is relatively high compared to others since it is a common valve condition (between 2–3% of the population presents a significant TR) [1, 2, 17, 36]. However, although some causes may favor an association between both VHDs, such as in degenerative diseases, the pathophysiology of AS itself is the probable factor in TR development [13, 40]. Ventricular secondary etiology is the most common cause in cases of left-sided VHDs, especially in the advanced stages of the illness. This is secondary to the generation of post-capillary pulmonary hypertension and subsequent right overload with the dilation or dysfunction of the right ventricle, which is described in 18–54% of cases (Fig. 3) [1, 25, 41]. Furthermore, MR development may also promote the presence of TR in this context [1, 13]. Nonetheless, other causes, such as the presence or development of atrial fibrillation, may also lead to the presence of TR in this setting because of increased atrial pressures [26, 40].

In terms of prognosis, in cases with moderate-severe or higher untreated TR who underwent intervention for severe AS, several studies have reported higher mortality at 30 days, especially at the one-year follow-up [1, 38, 39, 41, 42, 43, 44]. However, when performing the pertinent statistical analysis, most of them suggest that TR may not be the direct cause of this increased mortality and could instead be an associated factor [1, 39, 42, 44]. Thus, although significant TR is related to worse prognosis, there is currently not enough evidence to confirm that this is the reason for the increased mortality in most cases.

## 3. Key Points in Current Treatment Recommendations

Although studies are consistent on the potential negative prognostic effect of AS associated with other significant VHDs, each case has no consensus on the best treatment [5, 6]. This is partly because the risk of serious complications and perioperative mortality rises as the complexity of the intervention increases. In addition, many percutaneous treatments for TR and MR have not yet clearly demonstrated their clinical benefit in terms of mortality [5, 6, 7, 8, 9]. Therefore, any decision on the best treatment in each clinical scenario is not well-established, especially in borderline cases, which remain among the most frequent. Furthermore, the secondary functional component in MR and TR plays a vital role and can potentially be improved with AS treatment. Thus, there are concerns about whether single AS treatments would avoid the use of more complex surgical or double interventions [1, 2].

**Fig. 4.**
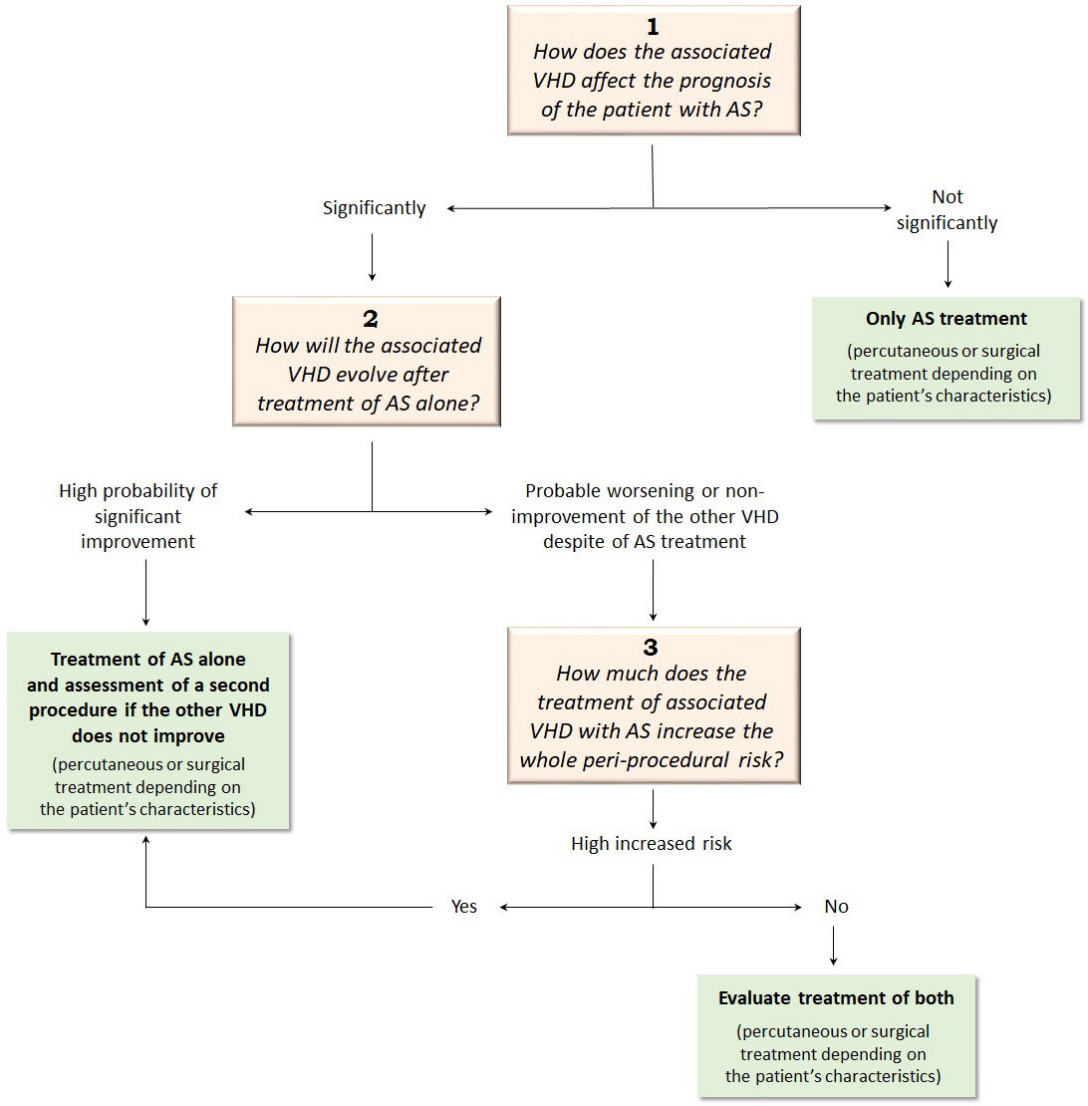
**Potential management pathway for severe AS associated with other VHDs**. AS, aortic stenosis; VHD, valvular heart disease.

Three questions should be raised in the management of severe AS associated with other VHDs before deciding on the type of intervention (Fig. 4). The first is how the additional VHD affects the prognosis of the AS patient. If the prognosis is not significantly altered or there is no clear scientific evidence, then a single treatment should be appropriate. The second is how the additional VHD will change after applying the single AS treatment. If there is potential for it to be significantly improved by its predominant mechanism, then a single treatment may again be the most suitable. Finally, it is necessary to assess how much the added treatment for the other VHDs increases the risk of the intervention. This should be evaluated according to the risk scales but should also consider the possibilities of each center [5, 6]. In this scenario, the role of the Heart Team is essential in determining the right treatment option in each case.

### 3.1 Global Key Points in the Assessment of AS Treatment with Other VHDs

In patients aged ≥75 years or with high interventional risk (STS-PROM/EuroSCORE IIf >8%) or in whom it is not possible to surgically treat the other VHD, according to clinical practice guidelines, the recommendation is to perform TAVR as the first option [5, 6]. Subsequently, depending on the patient’s evolution, a percutaneous technique may be considered to treat the other VHD [1, 2]. Although some authors propose that both treatments be performed during the same procedure, this combination seems to substantially lengthen the intervention and increase the potential of secondary complications [3, 4, 5]. Moreover, performing a two-stage procedure allows change assessments in the other VHD, as in cases of MR and TR, which can result in significant decreases as the cardiac hemodynamics improve [1, 2, 4].

Alternatively, in patients <75 years or with low surgical risk (STS-PROM/EuroSCORE IIf <4%), surgical treatment, whenever possible, is usually the best option if the clinical impact of the second VHD is significant [5, 6]. In addition, it is important to always consider the patient’s wishes in this decision-making process before opting for a specific treatment.

### 3.2 Specific Key Points in the Assessment of AS Treatment with MR

An accurate valvular assessment before intervention is mandatory in this scenario for the optimal management of mitral valve disease. It is crucial to perform a precise MR grading, with different imaging techniques, if necessary, to assess its hemodynamic impact [1, 5, 6]. Furthermore, the different parameters potentially associated with a likely persistence or worsening of the MR should also be assessed if it remains untreated (Table 1, Ref. [29, 30, 33, 34, 45, 46, 47]). Importantly, more than 70% and 60% of cases with severe and moderate MR, respectively, improve their regurgitation by one degree after aortic treatment [34]. This improvement in MR grade is also associated with a better clinical prognosis [34].

**Table 1.** **Potential risk factors before valve intervention are associated with the MR being stabilized or worsening at the subsequent follow-up**.

Potential risk factors during previous interventions	Cut-off point
Atrial fibrillation/flutter [30, 33, 34, 45, 46]	Presence before the procedure
Organic MR [33, 45]	Significantly associated pathology for the mitral valve leaflets, annulus, and chordate or papillary muscles
Mitral leaflet calcification [30]	Calcification degree 2 or 3 by MDCT (2 = nodules of calcification at both leaflets; 3 = extensive calcification at both leaflets, or restrictive calcification of one leaflet)
Mitral annular calcification [30]	Calcification degree 2 or 3 by MDCT (2 = focal calcification of less than 50% of the annulus but >1/3; 3 = >50% of annulus circumference calcified)
Mitral annular dilation [30]	>35.5 mm
Pulmonary hypertension [33, 45]	sPAP >55–59 mmHg*
Low baseline aortic gradient [33, 34]	<40 mmHg
Preserved ejection fraction [47]	≥50%
Unenlarged LVEDD [34, 47]	<50 mm
Unenlarged LVESD [47]	<36 mm
Prior aortic valve procedure [34]	-
Self-expanded valve implantation [29]	-

* The cut-off point is slightly different depending on the study. LVEDD, left ventricular end-diastolic diameter; LVESD, left ventricular end-systolic diameter; MDCT, multidetector computed tomography; sPAP, systolic pulmonary artery pressure; MR, mitral regurgitation.

Most studies conclude that the presence of atrial fibrillation or a flutter before the intervention is a risk factor associated with the non-improvement of MR after the procedure [30, 33, 34, 45, 46]. Moreover, evidence of significant pulmonary hypertension is another parameter for a poorer MR outcome [33, 45]. Others, such as organicity in the etiology of the lesion, valve calcification, dilation of the valve annulus, presence of a non-dilated left ventricle with preserved ejection fraction, low aortic transvalvular gradient, self-expanded valve implantation, or a previous history of aortic intervention have also been proposed as potential negative parameters in the evolution of MR [29, 30, 33, 34, 45, 47]. However, these studies are not conclusive on these, and although they should be considered in the pre-intervention assessment, none should be individually definitive when choosing the treatment. Thus, even though it is reported that a significant improvement in MR with AS treatment occurs in more than 60% of patients after the procedure [34], it is uncertain which cases will progress unfavorably if left untreated. The combined assessment of the above factors may help decide the best approach. Nevertheless, the technical feasibility of other potential therapies is also relevant should the prediction subsequently fail.

In high-risk patients with significant residual MR after the intervention, if feasible, surgical management of the aortic and mitral valves is usually the treatment of choice to avoid potential complications [5, 6]. When TAVR is performed, and subsequent MR worsening is detected, percutaneous mitral treatment with one of the current techniques, especially edge-to-edge therapy, is a potential second-step solution [3, 4, 5, 6]. Percutaneous implantable mitral prosthesis treatment can also be considered, although results in this context remain limited [3]. Finally, some authors also propose surgical treatment for MR after TAVR, should it be significant and the surgical risk not prohibitive [1]. Nevertheless, this scenario is rare nowadays since, in most cases, the decision to undergo TAVR involves either an advanced age or high surgical risk.

### 3.3 Specific Key Points in the Assessment of AS Treatment with MS

Concomitant mitral valve surgery is recommended for patients with mitral valve areas ≤1.5 ${\mathrm{cm}}^{2}$ when undergoing surgical AVR for severe AS [5, 6]. However, in those patients with high surgical risk, treatment with TAVR and mitral balloon valvuloplasty may be appropriate, especially in cases with rheumatic valvular disease [1, 5, 6]. Recently, the development of new percutaneous implantable prostheses through different vascular accesses has provided the opportunity for second-stage treatment in those who require it and whose anatomy is suitable [48, 49, 50]. Nevertheless, few candidates are currently eligible for this type of prosthetic valve due to the specific requirements that must be met for it to be implanted [51]. Moreover, to date, the results obtained by these treatments are limited, and in most clinical scenarios, when evaluated, the likelihood of significant subsequent complications is high, as in cases with severe mitral annulus calcification [51].

### 3.4 Specific Key Points in the Assessment of AS Treatment with TR

Currently, surgical treatment of the tricuspid valve in the AS setting is based on TR severity and valvular annulus size [5, 6]. If the valvular annulus is dilated (≥40 mm or >21 mm/$m^{2}$ by two-dimensional (2D) echocardiography), acting on the tricuspid valve is advised, even if TR is mild. This recommendation is motivated by two concerns: the poorer clinical prognosis of patients with significant residual TR and/or with secondary dilated annulus post-intervention and the high surgical risk of a potential second procedure [52, 53]. Therefore, if the interventional risk is not particularly high and contraindications are absent, surgical treatment of both lesions is usually the first choice [5, 6]. Furthermore, it should be noted that the percutaneous treatment of TR has currently shown symptomatic improvement in selected patients. However, its benefit on mortality in this clinical scenario has yet to be established [54, 55, 56].

If TAVR is chosen, it is relevant to highlight that 30–60% of cases significantly improve TR, with more than half of them exhibiting a normalized right ventricular function [39, 42, 43, 57]. The potential risk parameters for poor outcomes of untreated TR are not clearly defined in this context. This is partly because several studies suggest that residual TR could be a factor associated with others that would determine the poor prognosis of those cases, such as right ventricular dysfunction/dilation, pulmonary hypertension, or dilation of the tricuspid valve annulus [1, 39, 42, 44, 58]. However, some authors have reported that the presence of atrial fibrillation at baseline, significant tricuspid annular dilation (>25 mm/$m^{2}$), and at least a moderate post-TAVR aortic regurgitation may be independent risk markers for a poorer TR after the intervention has been performed [59, 60, 61].

In the case of the subsequent worsening of TR, performing the percutaneous tricuspid procedure as a second-stage intervention seems to be the best approach [1, 5, 6, 62]. Edge-to-edge therapy has the most accumulated experience [63]. However, multiple implantable devices and prostheses are currently available for percutaneous TR treatment, although the experience and benefit of each remain very limited [64, 65]. Thus, valvular assessment and the individualized study of each case will determine the best device according to the clinical and anatomical characteristics [63]. Conversely, tricuspid surgery following TAVR has also been raised as a potential option [1]. Nevertheless, as is the case for MR, whereby the indication for TAVR usually involves elderly and surgically high-risk patients, very few candidates can be included in this group.

## 4. Conclusions

The management of severe AS in the context of other significant VHDs has changed considerably over recent years. The range of treatment possibilities has expanded with the emergence of multiple devices, mainly percutaneous. However, there is currently no consensus on the best approach for each case. Therefore, the Heart Team’s decision at each center, an accurate valvular analysis before the intervention, and the patient’s wishes remain mandatory in determining the appropriate approach. This valvular analysis should be considered to determine how the additional VHDs will affect a patient’s prognosis, how it will evolve in cases of single AS treatments, and the added risk of treating both lesions in the same procedure. Nevertheless, given the expected technological developments in this field, many of the current decision-making paradigms are likely to change considerably in the coming years.

## Abbreviations

AS, aortic stenosis; MR, mitral regurgitation; MS, mitral stenosis; TAVR, transcatheter aortic valve replacement; TR, tricuspid regurgitation; VHD, valvular heart disease.
